# Diffusion tensor imaging with free‐water correction reveals distinctions between severe and attenuated subtypes in Mucopolysaccharidosis type I

**DOI:** 10.1002/jimd.12830

**Published:** 2025-01-06

**Authors:** Alena Svatkova, Ofer Pasternak, Julie B. Eisengart, Kyle D. Rudser, Petr Bednařík, Bryon A. Mueller, Kathleen A. Delaney, Elsa G. Shapiro, Chester B. Whitley, Igor Nestrašil

**Affiliations:** ^1^ Department of Pediatrics, Medical School University of Minnesota Minneapolis Minnesota USA; ^2^ Danish Research Centre for Magnetic Resonance, Centre for Functional and Diagnostic Imaging and Research Copenhagen University Hospital Amager and Hvidovre Copenhagen Denmark; ^3^ Department of Radiology, Centre for Functional and Diagnostic Imaging and Research Copenhagen University Hospital Amager and Hvidovre Copenhagen Denmark; ^4^ Departments of Psychiatry and Radiology Brigham and Women's Hospital, Harvard Medical School Boston Massachusetts USA; ^5^ Division of Biostatistics and Health Data Science, School of Public Health University of Minnesota Minneapolis Minnesota USA; ^6^ Department of Psychiatry & Behavioral Sciences University of Minnesota Minneapolis Minnesota USA; ^7^ Present address: BioMarin Pharmaceutical San Rafael California USA

**Keywords:** attenuated MPS, diffusion tensor imaging (DTI), free‐water, Hurler syndrome, Mucopolysaccharidosis type I, perivascular Virchow Robin spaces

## Abstract

Mucopolysaccharidosis type I (MPS I) is an inherited lysosomal storage disorder leading to deleterious brain effects. While animal models suggested that MPS I severely affects white matter (WM), whole‐brain diffusion tensor imaging (DTI) analysis was not performed due to MPS‐related morphological abnormalities. 3T DTI data from 28 severe (MPS IH, treated with hematopoietic stem cell transplantation—HSCT), 16 attenuated MPS I patients (MPS IA) enrolled under the study protocol NCT01870375, and 27 healthy controls (HC) were analyzed using the free‐water correction (FWC) method to resolve macrostructural partial volume effects and unravel differences in DTI metrics accounting for microstructural abnormalities. FWC analysis in MPS IH compared to HC revealed higher free‐water fraction (FWF) in all WM regions with increased radial (RD) and mean diffusivity (MD). Higher RD, MD, and FWF in cingulate and FWF in temporal WM were observed in MPS IA relative to HC. FWF and RD in the corpus callosum (CC) were higher in MPS IH than in MPS IA. Reaction time was correlated with fractional anisotropy (FA) in frontal and parietal WM in MPS IH. FA in temporal and central WM correlated with d‐prime in MPS IA. The HSCT age was related to FA in parietal WM and FWF in frontal WM in MPS IH. FWC delineated subtype‐specific WM microstructural abnormalities linked to myelination that were more extensive in MPS IH than IA, with CC findings being a key differentiator between subtypes. Earlier age at HSCT was related to preserved WM microstructure in the brain of MPS IH patients. Free water‐corrected DTI distinguishes severe and attenuated MPS I patients and reveals a relationship between attention, age at HSCT, and white matter microstructure.

**Key Points**

- DTI analysis in MPS is challenging due to morphological brain abnormalities.
- FWC eliminates the effect of higher free‐water content.
- Severe and attenuated MPS I subtypes exhibit distinct spatial patterns of WM damage.
- Distinct phenotypes and treatment strategies likely cause WM differences.
- HSCT at an earlier age preserves WM microstructure in severe MPS I patients.

## 1 INTRODUCTION

Mucopolysaccharidosis (MPS) type I is an inherited lysosomal storage disease caused by a deficiency of α‐L‐iduronidase enzyme, in which pathological glycosaminoglycans (GAG) accumulation leads to severe multi‐organ damage.^1^ Murine models have shown that increased lysosomal GAG storage in the brain results in astrocytosis and microgliosis as well as axonal dystrophy, abnormal dendritic sprouting, enlargement of the axonal hillock, alteration of synaptic proteins, and myelin membrane damage.^2^, ^3^ Chronic cellular brain damage and neuroinflammation^2^ further promote inflammatory processes, leading to a vicious circle.^4^, ^5^ Myelin alteration and demyelination due to GAG buildup have been depicted in animal models using diffusion tensor imaging (DTI),^6^, ^7^, ^8^ direct‐myelin mapping technique,^9^ brain morphometry,^10^ and single‐voxel spectroscopy.^11^ However, to date, whole‐brain DTI analysis in MPS patients has not been successfully performed due to the presence of structural MPS‐related brain abnormalities.

A single pilot DTI study manually delineating corpus callosum (CC) in severe (MPS IH, Hurler syndrome) and attenuated (MPS IA, Hurler‐Scheie and Scheie syndrome) MPS I patients suggested that the treatment and disease contribute to subtype‐specific white matter (WM) characteristics and may potentially predict neuropsychological performance.^12^ Current MPS I treatment standards offer hematopoietic stem cell transplantation (HSCT) to MPS IH patients who face rapid cognitive decline without treatment and intravenous or intrathecal enzyme replacement therapy (ERT) to MPS IA patients,^13^, ^14^, ^15^, ^16^ in whom neurocognitive symptoms broadly vary.^15^ Emergent treatment strategies, such as intrathecal ERT, substrate reduction therapy, receptor‐mediated (“Trojan horse”) enzyme delivery to the brain, or gene therapies,^13^, ^17^ addressing HCST and ERT limitations substantiate the need to evaluate WM alterations in MPS subtypes and provide noninvasive, reliable MRI outcomes that are quantitative and thus well‐suited for clinical trials.

Brain atrophy, communicating hydrocephalus with ventriculomegaly, and dolichocephalic head shape^18^ in MPS patients indeed limit the utilization of group analysis techniques that employ brain MRI normalization to a standard space.^19^ Hence, the aforementioned single DTI study in MPS patients remained limited to manual region‐of‐interest (ROI) selection of the CC^12^ and, thus, did not provide whole‐brain WM analysis. Also, the partial volume effect due to atrophy and cystic enlarged perivascular spaces (PVS)^18^, ^20^ in MPS brains biases DTI measures by artificially decreasing fractional anisotropy (FA) and elevating diffusivity values such as axial and radial diffusivity.^21^

Thus, the free‐water correction (FWC) method was employed to diminish the effect of enlarged PVS and brain atrophy on DTI measures.^22^ FWC quantifies the free‐water fraction (FWF) as a proxy for the extracellular space volume and estimates diffusivity measures from water molecules in the proximity of tissue to derive free‐water corrected (FWC) DTI measures.^22^ FWC and techniques that analyze WM in subject‐space^23^ were utilized to delineate microstructural WM deficits between severe MPS IH, attenuated MPS IA subtypes, and healthy individuals in an automated, unbiased manner.

We investigated diffusion properties and FWFs using a whole‐brain analytical approach and compared the severity of WM alterations between MPS IH, MPS IA, and HC. WM values were then related to neuropsychological measures in MPS IH and MPS IA subtypes.

## 2 MATERIALS AND METHODS

### 2.1 Study participants

Study participants with MPS I were enrolled in the longitudinal protocol NCT01870375 of the Lysosomal Disease Network (Longitudinal Studies of Brain Structure and Function) with the following inclusion criteria: a confirmed diagnosis of MPS I, physical ability to undergo a one‐hour non‐sedated scan, and hearing and vision adequate for neuropsychological testing. Eligible participants had no ventricular shunt.^24^

Forty‐four patients with MPS type I—28 severe MPS I (MPS IH) (mean age 11.6, range of 2.6–24.5 years; 14 males/14 females) and 16 attenuated MPS patients (Hurler–Scheie or Scheie, MPS IA; mean age 15.6, range of 7.1–22.5 years; 10 males/6 females) and 27 HC (mean age 7.4, range of 5–10 years; 11 males/16 females) were analyzed. Diffusion data of one male MPS IH subject (age 12.6 years) was excluded due to severe movement artifacts. Those younger than 4 years (5 MPS IH patients) were excluded from the analysis due to the rapid ongoing brain development before age four, which may bias the measurements. 27 out of 28 MPS IH patients underwent an HSCT at a mean age of 1.44 ± 0.74 years of age (14 males/13 females). 17 MPS IH patients received bone marrow transplants (4 from unrelated matched subjects, 13 from a sibling, and 1 from an unknown resource) and 10 cord blood transplants (9 from unrelated matched subjects and 1 from an unknown source).

Five patients from the MPS IA group have more severe L238Q genetic mutation (mean age 17.5, range of 16–20, 3 males/2 females).^25^ Healthy children (HC) with no history of developmental delays or neurological and psychiatric disorders were used as a control group.

Since the patient and control groups in the study varied in age range, we performed a post hoc analysis within the same age range. Therefore, for this analysis, we excluded MPS I patients younger than 4 and older than 13 years of age from the cross‐sectional analysis, resulting in the cohorts of MPS IH (*n* = 17; mean age 8.9, 4.7–12.9 years; 7 males/10 females) and MPS IA (*n* = 5; mean age 9.6, 7.1–12.7 years; 4 males/1 female) that were compared versus HC. Lastly, in another analysis, we included only patients older than 7 and younger than 23 years of age for a post hoc comparison of MPS IH (*n* = 16, 6 males/10 females, mean age 11.6, 7.5–17) versus MPS IA patients (*n* = 16; 10 males/6 females, mean age 15.6, 7.1–22.5).

### 2.2 Standard protocol approvals, registrations, and patient consent

Study participants were recruited in the University of Minnesota IRB‐approved studies (IRB protocol ID 0905 M65804—PI Shapiro, STUDY00014153—PI Whitley, 1202 M10383—PI Nestrasil). All participants, parents, and/or legal guardians gave signed informed consent; assent was obtained from children and those over 18 years with a legal guardian, which included permission to share de‐identified data with the RDCRN (Rare Disease Clinical Research Network) Data Monitoring and Coordination Center.

### 2.3 MRI data acquisition and analysis

Diffusion MRI data were acquired on 3 T Trio Siemens scanner with a 12‐channel RF head coil (Siemens, AG, Erlangen, Germany) at the University of Minnesota Center for Magnetic Resonance Research (CMRR) using 12 diffusion‐weighted (*b* = 1000 s/mm^2^) and 1 unweighted (*b* = 0 s/mm^2^) images, NEX = 3, TR = 8500 ms, TE = 90 ms, FOV = 256 × 256, voxel size = 2 × 2 × 2 mm^3^, 64 slices. Gradient echo (GRE) fieldmap was acquired using TR = 700 ms, TE1/TE2 = 4.62/7.08 ms, FOV = 256 × 256, voxel size = 2 × 2 × 2 mm^3^, 64 slices. Anatomical 3D T1 MPRAGE scans were acquired using TR = 2530 ms, TE = 3.65 ms, TI = 1100 ms, and voxel size = 1 × 1 × 1 mm^3^.

WM regions were segmented from the anatomical MRI using Freesurfer version 5.3 (FS)^26^ based on individual cortical parcellation from Desikan‐Killiany atlas.^27^ Results from automated FS segmentation were visually checked for possible errors. Omissions of the FS algorithm were corrected using standard procedures recommended in the FS manual. More on the FS methodology and volumetric results are presented in our previous publication.^24^

Diffusion MRI data were visually inspected and then preprocessed using FSL version 5.0.6 (FMRIB Software Library, http://www.fmrib.ox.ac.uk/fsl)^28^ and DTIPrep.^29^ Firstly, data were corrected for possible geometric distortions and field inhomogeneities using fieldmap scans and skull‐stripped using FSL. Comprehensive correction of diffusion‐related artifacts such as eddy currents, head motion, bed vibration and pulsation, venetian blind artifacts, and slice‐wise and gradient‐wise intensity inconsistencies was done using the DTIPrep pipeline.^29^

The free water imaging (FWI) algorithm was applied per subject, resulting in FWF maps and free‐water corrected (FWC) DTI data.^22^ The FWC DTI data included fractional anisotropy (FA_FWC_), mean diffusivity (MD_FWC_), axial diffusivity (AD_FWC_), and radial diffusivity (RD_FWC_) maps.

**FIGURE 1:**
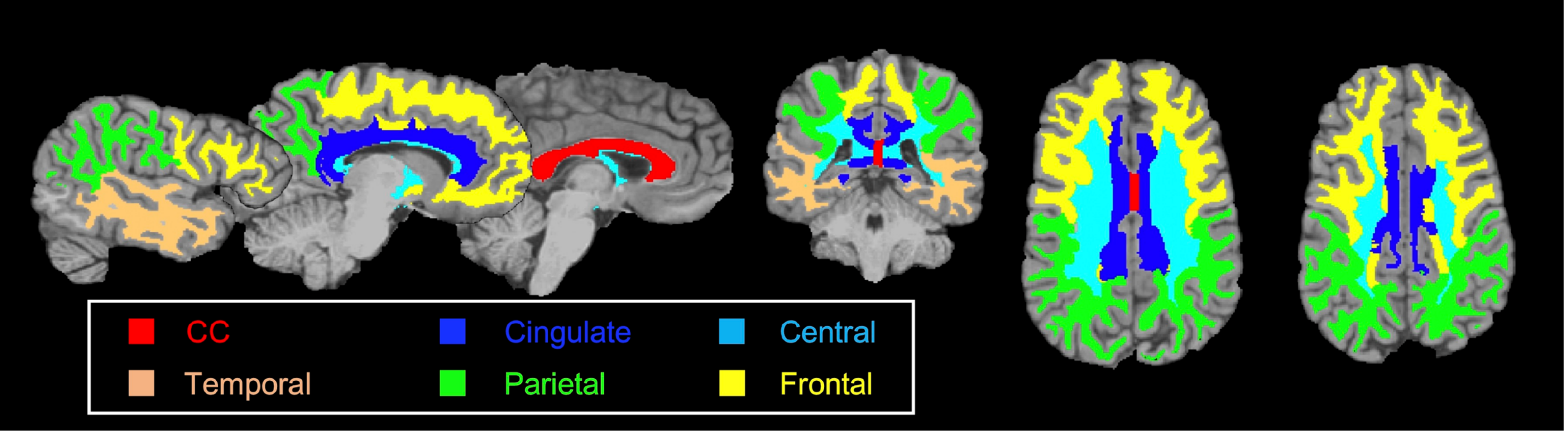
Regions of interest used in the DTI analysis. Masks identifying the regions of interest are visualized on T1‐weighted anatomical scans.

The Boundary‐based (BB)^23^ registration algorithm in FS was used to co‐register diffusion data and T1‐weighted images using FS segmentation to improve registration accuracy. The results of registration were visually verified. Regions‐of‐interest (ROIs) were concatenated based on the lobar anatomy into bilateral frontal, parietal, temporal, cingulate WM, and CC, and central WM regions (Figure 1). FWC maps were additionally weighted to account for atrophy level and the number of PVS spaces, which were larger than the voxel size using the formula. Weighting was done for all ROIs separately to calculate the final values of relevant diffusion measures using the following formula,

$${\mathrm{FA}}_{\text{final}}=\frac{1}{n}\;\Sigma \left({\mathrm{FA}}_{\mathrm{FWC}}\times \left(1-\mathrm{FWF}\right)\right)$$

where *n* is the number of voxels in the specific ROI. This was applied to all relevant diffusion measures to quantify averaged bilateral FA_final_, MD_final_, AD_final_, RD_final_, and FWF values from the cingulate, frontal, parietal, temporal, CC, and central WM.

### 2.4 Neuropsychological evaluation

A trained psychologist or psychometrist acquired scores on the Tests of Variables of Attention (TOVA) to assess attention and impulse control in patients and HC. The TOVA is a 22‐minute test of attention in which geometric targets are randomly presented, and a subject presses a switch when a target is presented and inhibits response for the non‐target. For participants younger than 5.5 years of age (i.e., 2 MPS IH patients), a shorter 10.8‐minute version was performed.^30^ Response Time (RT) and RT Variability (measurement of responding speed consistency), together with Errors of commission (measurement of behavioral dis‐inhibition and impulsivity) and omission (measurement of inattention) rates, were recorded. D‐Prime Score, which reflects the hit rate ratio to the “false alarm” rate, was calculated to determine perceptual sensitivity.^31^

Full‐scale IQ, performance, and verbal IQ were acquired using the Wechsler Scale of Intelligence standardized for a specific age range: using Wechsler Preschool and Primary Scale of Intelligence‐III (WPPSI‐III)^32^ for 4–6‐year‐old participants and Wechsler Abbreviated Scale of Intelligence (WASI) for older than 6 years.^33^ All tests were standardized for the participants' ages.

### 2.5 Statistical analysis

Comparisons FWF and FWC DTI values were adjusted for age as a potential confounding variable due to age strongly affecting WM microstructure.^34^ This approach was applied in primary and additional age‐restricted post hoc analyses of participants aged 4–13 years. The MPS IH and MPS IA comparative analysis was performed on the whole sample and on the more balanced cohorts with age‐restricted between 7 and 23 years of age as a post hoc analysis.

All analyses were performed for bilateral frontal, temporal, parietal, cingulate, and central WM regions and the CC. All tests were two‐tailed, and results are reported as p‐values. The multiple comparison corrected p‐value threshold was set to 0.0005 (Bonferroni correction 0.05/(90 test—5 DTI_final_ values × 6 regions × 3 between‐groups comparisons) = 0.00055) and only results with p‐value lower than 0.00055 are considered significant (Table 1, Figure 3).

**TABLE 1:** Differences between MPS IH, MPS IA, and healthy controls (HC).

		MPSI A versus MPS IH		MPS IH versus HC		MPS IA versus HC	
		ORIG	Post hoc	ORIG	Post hoc	ORIG	Post hoc
AD	CC	0.217	0.165	0.015	0.025	0.276	0.095
	Central	0.781	0.805	0.066	0.235	0.156	0.172
	Cingulate	0.833	0.950	<0.0005*	0.003	<0.0005*	0.002
	Frontal	0.161	0.499	0.007	0.023	0.577	0.142
	Parietal	0.580	0.439	0.021	0.032	0.395	0.716
	Temporal	0.270	0.178	0.667	0.935	0.111	0.028
FA	CC	0.038	0.030	<0.0005*	0.006	0.101	0.977
	Central	0.895	0.416	0.218	0.048	0.359	0.395
	Cingulate	0.021	0.008	0.004	0.014	0.987	0.355
	Frontal	0.595	0.791	0.530	0.194	0.396	0.757
	Parietal	0.893	0.690	0.063	0.045	0.235	0.658
	Temporal	0.235	0.248	0.008	0.003	0.451	0.778
FWF	CC	<0.0005*	<0.0005*	<0.0005*	<0.0005*	0.031	0.285
	Central	0.828	0.749	<0.0005*	<0.0005*	0.028	0.018
	Cingulate	0.017	0.026	<0.0005*	<0.0005*	<0.0005*	<0.0005*
	Frontal	0.322	0.758	<0.0005*	<0.0005*	0.009	0.038
	Parietal	0.648	0.683	<0.0005*	<0.0005*	0.001	0.001
	Temporal	0.873	0.501	<0.0005*	<0.0005*	<0.0005*	<0.0005*
MD	CC	0.001	0.002	<0.0005*	<0.0005*	0.006	0.042
	Central	0.876	0.964	0.001	0.003	0.058	0.048
	Cingulate	0.093	0.144	<0.0005*	<0.0005*	<0.0005*	<0.0005*
	Frontal	0.127	0.528	0.001	0.002	0.167	0.066
	Parietal	0.454	0.342	<0.0005*	<0.0005*	0.202	0.261
	Temporal	0.286	0.150	0.323	0.528	0.040	0.011
RD	CC	<0.0005*	<0.0005*	<0.0005*	<0.0005*	0.001	0.073
	Central	0.975	0.856	<0.0005*	<0.0005*	0.026	0.028
	Cingulate	0.018	0.034	<0.0005*	<0.0005*	<0.0005*	<0.0005*
	Frontal	0.112	0.574	<0.0005*	<0.0005*	0.027	0.034
	Parietal	0.296	0.279	<0.0005*	<0.0005*	0.061	0.132
	Temporal	0.330	0.142	0.137	0.265	0.015	0.005

*Note*: Comparisons were performed two‐tailed with a critical multiple comparison corrected *p*‐value <0.0005 reported using asterisk.

Table 2 summarizes standardized mean IQ and TOVA values, and their differences between groups were evaluated using an independent sample t‐test. Since cognitive tests are already standardized for age, these analyses did not include age as a covariate.

**TABLE 2:** Cognitive and attentional scores.

		MPS IH	MPS IA		MPS IH versus MPSIA	MPS IA without L238Q versus with L238Q
		Mean (SD)	Mean (SD)			
			MPS IA without L238Q	MPS IA with L238Q	Mean difference (95% CI)	Mean difference (95% CI)
			Mean (SD)	Mean (SD)	*p*‐value	*p*‐value
IQ	FSIQ	83.19 (15.45)	91.33 (19.97)		−8.1 (−20.1;3.8)	31.1 (15.2; 47.0)
			101.70 (15.04)	70.60 (8.96)	0.177	<0.001
	VIQ	82.19 (14.58)	91.27 (16.77)		−9.1 (−19.7; 1.6)	24.4 (9.9;38.9)
			99.40 (12.37)	75.00 (11.98)	0.093	0.003
	PIQ	84.05 (15.37)	90.27 (18.89)		−6.2 (−17.8; 5.4)	29.2 (17.1; 41.3)
			100.00 (14.78)	70.80 (6.83)	0.284	<0.001
TOVA	D‐prime	69.65 (9.46)	82.14 (25.58)		−12.7 (−27.5; 1.9)	25.8 (−1.6; 53.2)
			91.00 (24.78)	65.20 (18.81)	0.085	0.062
	Omission error	51.58 (17.40)	80.8 (33.25)		−29.2 (−48.0; −10.4)	39.9 (6.8; 7.9)
			94.10 (30.65)	54.20 (20.72)	0.006	0.022
	Commission error	74.00 (28.52)	91.80 (34.26)		−17.8 (−40.5; 4.9)	18.0 (−22.7; 58.7)
			97.80 (36.68)	79.80 (28.49)	0.119	0.356
	Response time	79.71 (19.07)	106.53 (34.93)		−26.8 (−46,8; −6.8)	22.1 (−19.8;64.0)
			114.20 (37.24)	91.20 (26.61)	0.01	0.275
	Variability	61.70 (19.53)	91.13 (35.81)		29.4 (−49.9; −8.9)	23.0 (−17.6;63.6)
			114.20 (37.24)	91.20 (26.61)	0.006	0.243

*Note*: Values for MPS IH and MPS IA, including MPSIA without and with L238Q mutation, are summarized as means and standard deviations (SD). Between‐group comparisons between MPS IH and MPS IA, as well as MPS IA with and without the L238Q mutation, were performed using an independent sample t‐test, and differences are reported as mean differences, 95% confidence intervals (Diff (95% CI)), and *p*‐values.

Separate regression analyses were conducted in MPS IH and IA, with separate analyses performed for MPS IA patients with and without the L238Q mutation. Neuropsychological measures were set as the dependent variable and averaged FWI metrics as the independent variable to determine the association of WM parameters with altered neuropsychological outcomes within each of these MPS subtypes. Regression analyses were conducted for the cingulate, frontal, temporal, parietal, central WM, and CC to evaluate the relationship between neuropsychological variables and specific WM regions. In MPS IH, the relationship between the age at HSCT and FWI was calculated using FWI metrics as dependent variables and the age at HSCT and the actual age at the MRI scan as independent variables. The multicollinearity (i.e., high correlation between independent variables) was checked in the regression models.

All regression analyses were considered exploratory and were not corrected for multiple comparisons. However, the results with critical p‐values <0.01 were considered significant. All analyses were performed with SPSS 25.0.0 (SPSS Inc., Chicago, IL, USA).

## 3 RESULTS

### 3.1 Differences between MPS IH and HC

**FIGURE 2:**
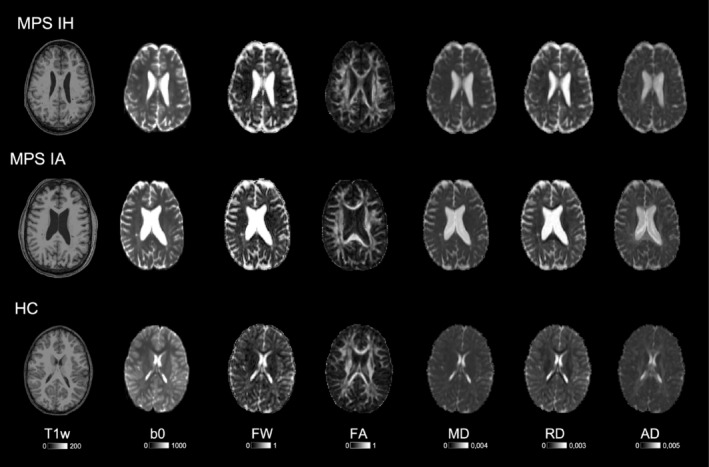
Example MRI data of MPS IA, MPS IH patients, and healthy control (HC). Showing anatomical T1‐weighted (T1w), diffusion‐weighted scans (b0), free‐water fraction (FWF), and free‐water corrected diffusion maps of fractional anisotropy (FA), mean (MD), radial (RD), and axial diffusivity (AD).

Both primary and post hoc FWC‐weighted analyses with age as a covariate consistently showed significantly higher FWF in the CC, frontal, temporal, parietal, cingulate, and central WM of MPS IH patients compared to controls (Table 1, Figure 2). RD_final_ was significantly higher in the same WM areas except for the temporal lobe, while MD_final_ was higher in the CC, cingulate, and parietal WM. Significantly higher AD_final_ in the cingulate and lower FA_final_ in the CC remained trend‐level significant in the post hoc age‐matched analyses.

### 3.2 Differences between MPS IA and HC

**FIGURE 3:**
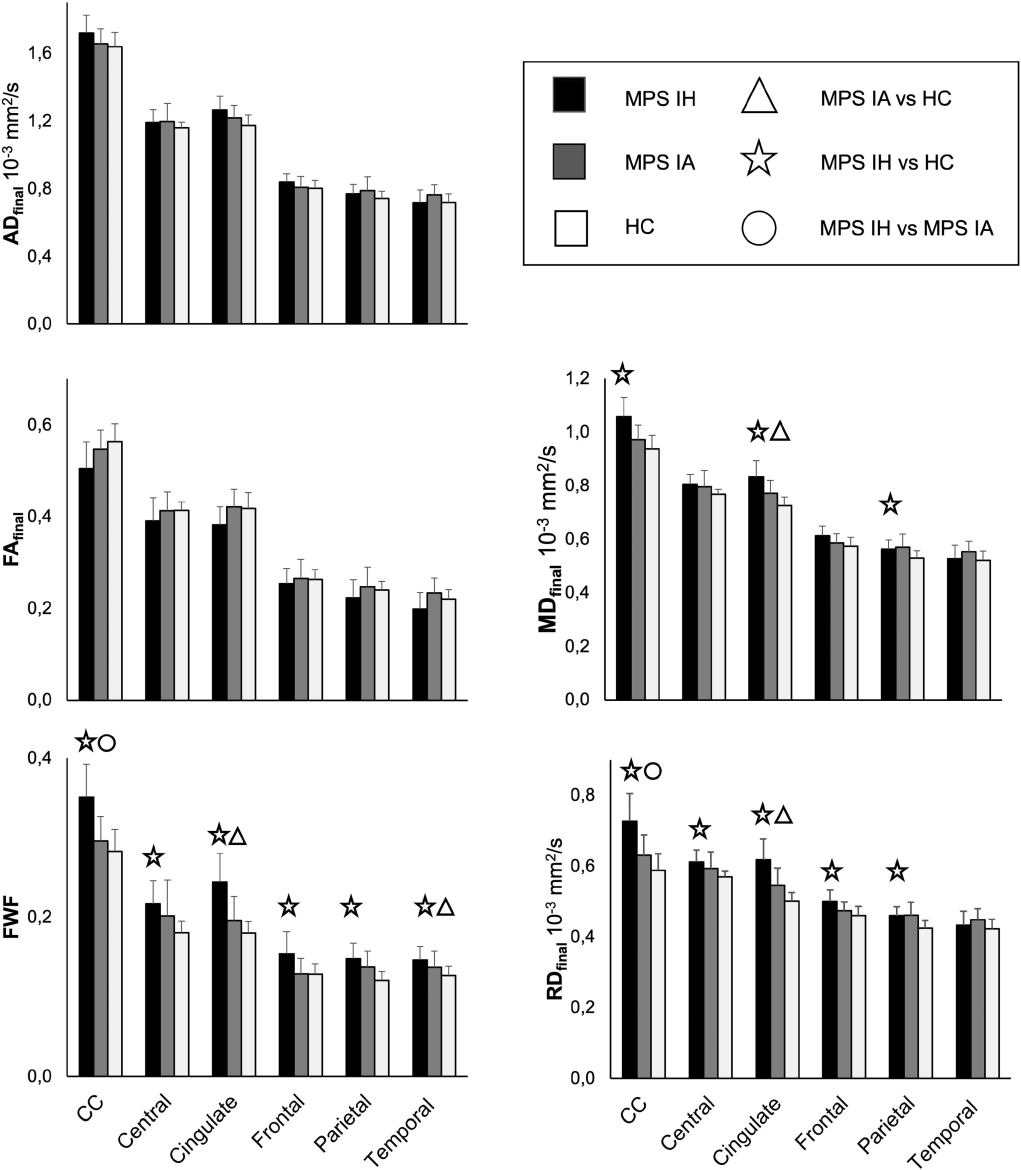
Group summaries of free‐water fraction (FWF), free‐water corrected (FWC) fractional anisotropy (FA_final_), axial diffusivity (AD_final_), mean (MD_final_), and radial diffusivity (RD_final_). Data are presented as mean ± SD. At *p* < 0.0005 significance level, the asterisk indicates the difference for MPS IH vs. healthy controls (HC), the triangle for MPS IA vs. HC, and the circle for MPS IH vs. MPS IA.

Age‐adjusted analyses on the original and age‐restricted groups detected higher MD_final_ underpinned by higher RD_final_ in cingulate WM and higher FWF in cingulate and temporal WM in MPS IA relative to HC. However, the significantly higher AD_final_ in cingulate WM did not reach the significance level in the post hoc analyses (Table 1, Figure 3).

### 3.3 Differences between MPS IH and MPS IA

Direct comparisons between the disease subtypes, adjusted for age in both primary and post hoc analyses, revealed significantly higher RD_final_ and FWF in the CC of MPS IH patients compared to MPS IA (Table 1, Figure 3).

### 3.4 Neuropsychological measures, age at hematopoietic cell transplantation (HSCT), and WM parameters

Between‐group comparisons revealed lower IQ scores in MPS IH compared to MPS IA; however, the difference was non‐significant. MPS IH patients scored significantly lower in TOVA scores in omission errors, RT, and variability than attenuated patients (Table 2). MPS IA with the L238Q mutation under‐performed in all TOVA and IQ scores compared to those without this mutation, with all IQ subscales and omission error TOVA subscale demonstrating statistically significant differences between groups (Table 2).

**FIGURE 4:**
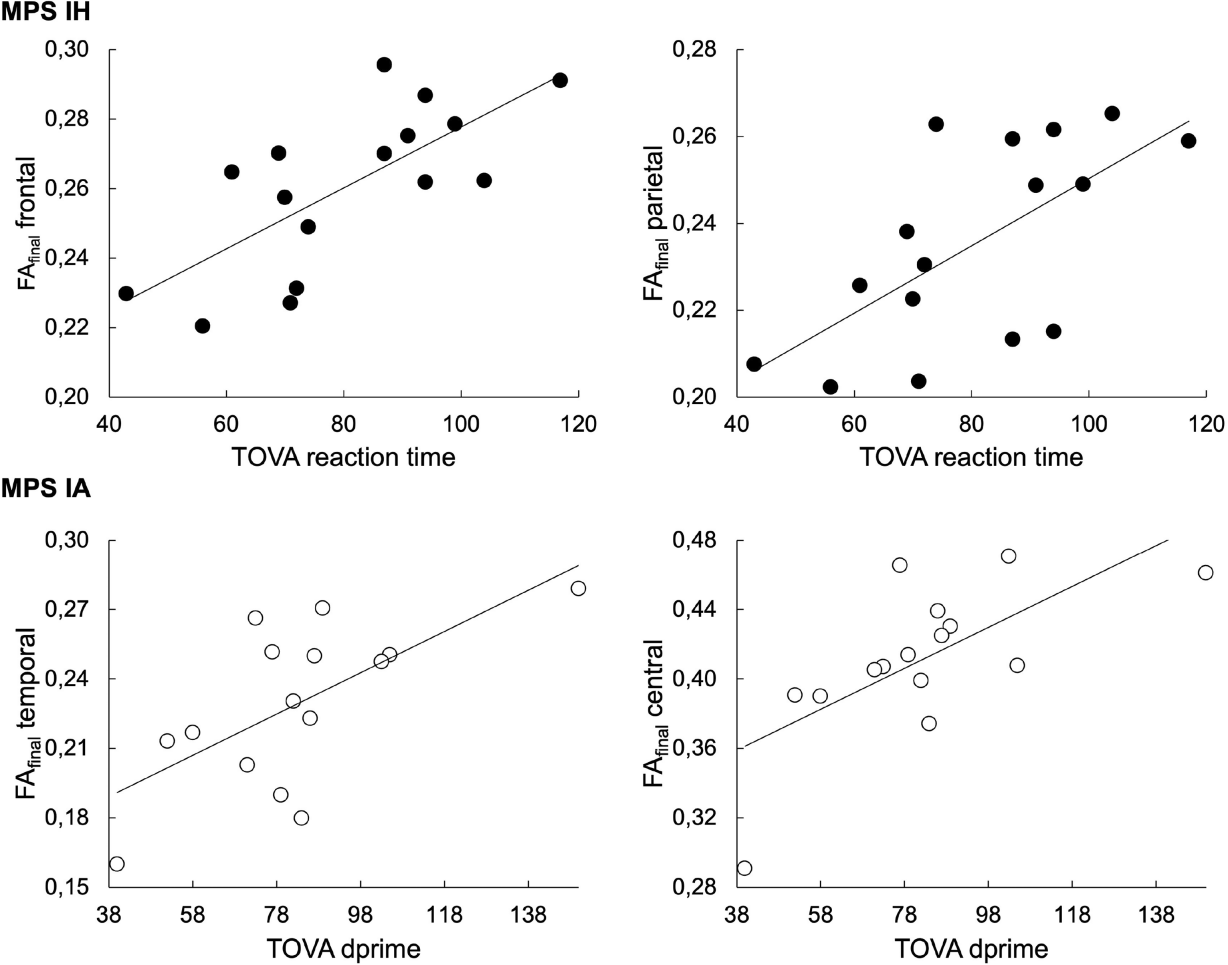
The association between diffusion and neuropsychological measures. Significant relationships (*p* < 0.01) between neuropsychological measures (attention measures) and averaged FA_final_ metrics in MPS IH (black circles) and MPS IA patients (white circles) are shown.

In MPS IH, regression analysis revealed a statistically significant relationship between reaction time and FA_final_ in the frontal (*p* = 0.002, *R* = 0.72; slope (95% CI) = 60 (27, 92)) and parietal WM (*p* = 0.006, *R* = 0.66, slope (95% CI) = 55 (19, 92)). Regression analysis of all MPS IA patients found a statistically significant relationship between d‐prime and FA_final_ in the temporal (*p* = 0.009, *R* = 0.65, slope (95% CI) = 48 (14, 81)) and central WM (*p* = 0.005, *R* = 0.68, slope (95% CI) = 40 (14, 65)) (Figure 4).

No statistically significant relationships (*p* < 0.01) were demonstrated between diffusion values and neuropsychological measures in MPS IA patients without the L238Q mutation. Regression analyses on five MPS IA patients with L238Q mutation were not performed because the small sample size limits the reliability and generalizability of these results.^35^

**FIGURE 5:**
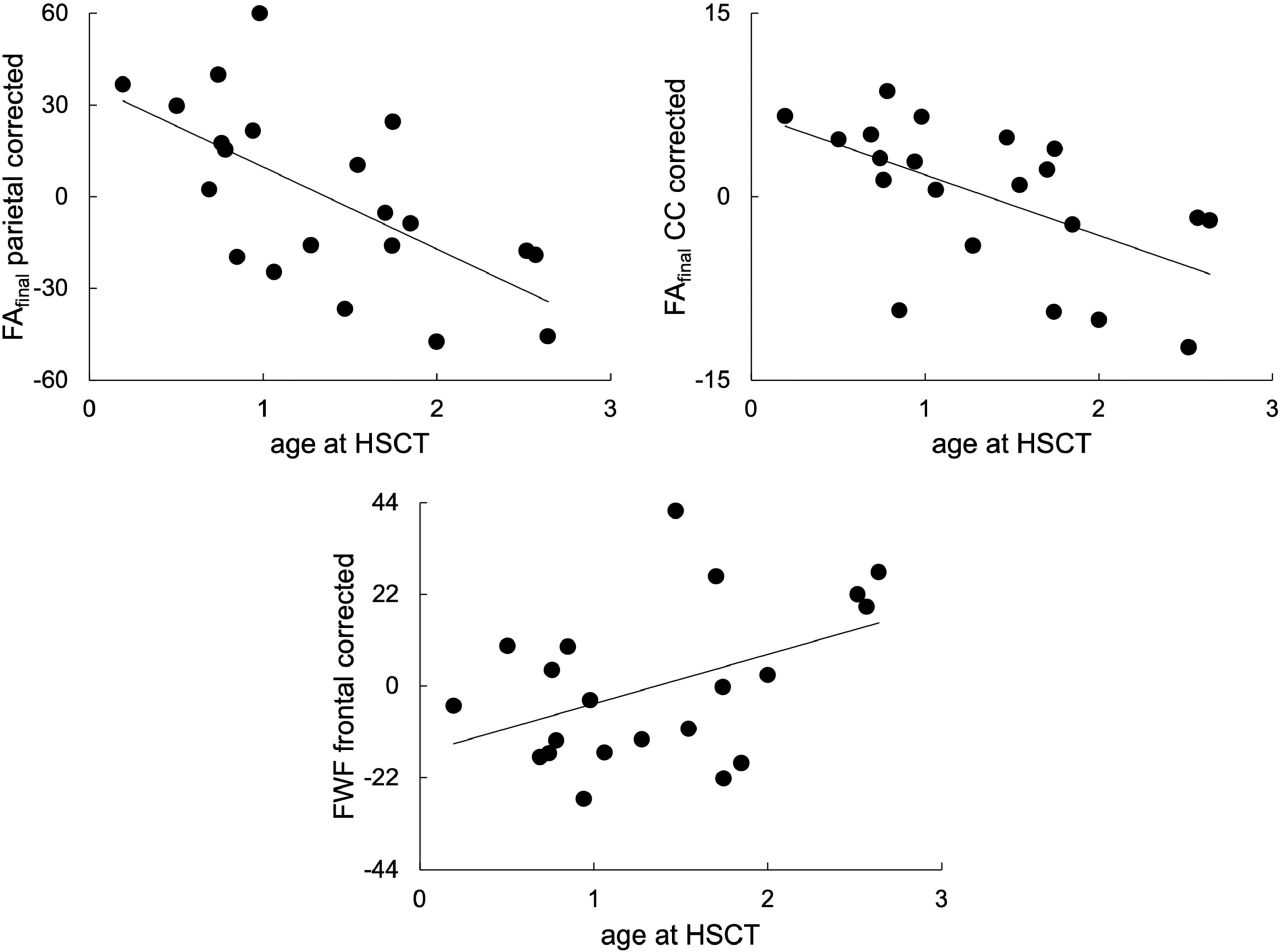
The relationship between diffusion measures corrected for actual age at the MRI scan and age at HSCT in MPS IH patients. Significant relationships (*p* < 0.01) between the age at HSCT and averaged age‐corrected FA_final_ and FWF metrics are shown.

Regression models in MPS IH found a statistically significant relationship between the age at HSCT and FA_final_ in parietal WM (*R*_overall model_ = 0.70, *R* ^2^_overall model_ = 0.49, *p*_overall model_ = 0.002; *p*_age at HSCT_ = 0.002, *R*_age at HSCT_ = −0.62; slope _age at HSCT_ (95% CI) = −27.5 (−43.3; −11.7); *p*_actual age_ = 0.16, *R*_actual age_ = 0.25, slope _actual age_ (95% CI) = 1.6 (−0.7; 4)). Additionally, a trend‐level significant relationship was observed in FA_final_ in the CC, driven by a significantly negative relationship between the age at HSCT and age‐corrected FA_final_ (*R*_overall model_ = 0.63; *R* ^2^_overall model_ = 0.40, *p*_overall model_ = 0.01; *p*_age at HSCT_ = 0.007, *R*_age at HSCT_= − 0.56, slope _age at HSCT_ (95% CI) = −50.6 (−85.7, −15.5); *p*_actual age_ = 0.05, *R*_actual age_ = 0.25, slope _actual age_ (95% CI) = −5.2 (−10.3, 0.02)). A significant pattern was found for FWF in frontal WM for the overall model with a trend‐level positive relationship between age‐corrected FWF and age at HSCT (*R*_overall model_ = 0.67; *R* ^2^_overall model_ = 0.45; *p*_overall model_ = 0.005, *p*_age at HSCT_ = 0.048, *R*_age at HSCT_= 0.38, slope _age at HSCT_ (95% CI) = 12.1 (0.1, 24.2); *p*_actual age_ = 0.012, *R*_actual age_ = −0.5, slope _actual age_ (95% CI) = −2.4 (−4.1, −0.6)) (Figure 5, Table S1). No significant multicollinearity was present between the age at the MRI scan and the HSCT age in the model.

## 4 DISCUSSION

Comparisons between MPS subtypes and healthy control participants revealed abnormalities in FWC‐weighted radial diffusivity (RD_final_), suggesting a preferential involvement of myelin deficits in MPS I brains.^36^ More profound alterations, characterized by higher RD_final_ and consequentially higher MD_final_, were detected in MPS IH compared to MPS IA in the CC. Compared to healthy individuals, MPS IH patients exhibited a more widespread alteration in FWF and RD_final_ than MPS IA. In MPS IA, higher FWF was limited to the cingulate and temporal WM, while the CC, frontal, parietal, and central WM affected in MPS IH were spared. Correlation analyses revealed a relationship between WM integrity and attention TOVA measures but not with IQ measures. Interestingly, attention spans were related to frontal and parietal WM metrics in MPS IH and temporal and central WM in MPS IA, suggesting an involvement of distinct brain areas in abnormal attention of MPS patients. In addition, the age at HSCT was inversely related to FA in CC and parietal WM while positively correlated to FWF in the frontal lobe, suggesting a positive impact of early HSCT on WM integrity in MPS IH patients.

The association between WM‐hyperintensities and myelination deficits in MPS was initially proposed by Gabrielli et al. (2004),^20^ and demyelination was detected by several studies utilizing various MRI approaches in animal MPS models.^8^, ^9^, ^15^ A recent murine study of the CC detected alteration in phosphoethanolamine levels and lower expression of myelin‐related genes in the tissue.^11^ This corroborated with conclusions from canine models, which confirmed a linear relationship between myelin‐binding protein and altered FA and RD, thus highlighting the critical involvement of demyelination in MPS.^8^ In agreement with electron microscopy findings,^2^ a novel MRI method for direct myelin mapping entitled Relaxation Along a Fictitious Field (RAFF) in the rotating frame of rank n (RAFFn), proved myelin density alteration in MPS I mice while showing an ability of sensitive MRI techniques to distinguish between normal and shiverer murine models.^9^ However, it is important to note that RD or FA changes, i.e., higher RD and lower FA, do not automatically imply a myelin lesion. Such findings may be caused by increased extracellular water content, e.g., vasogenic edema due to the blood–brain barrier disruption.^37^ The FWC aims to eliminate the effects of extracellular changes on the FA and RD, thus making it more specific to cellular and myelin changes.^22^

The destruction of WM was confirmed in an exploratory DTI study demonstrating alteration of FA and MD in the CC of MPS IH compared to MPS IA.^12^ However, the analysis only evaluated the CC and did not address unwanted effects of enlarged perivascular (PVS *aka* Virchow‐Robin) spaces on DTI metrics that alter FA values. Higher free‐water content in the brain of MPS IH patients with significant differences between MPS I subtypes in the CC suggests potential bias of previous DTI analyses in MPS I due to a higher level of extracellular water.^38^ While enlarged PVS also increase FWF, we weighted each diffusion measure by the actual tissue component to eliminate the effect of large PVS and atrophy on the analysis. Thus, our findings of FWF differences between MPS I subtypes are likely caused by elevated water in tissue or edema due to elevated GAG content or inflammation.^39^ While increased GAG content in perivascular regions, meninges, microglia, and neurons in MPS brains indeed causes prolonged neuro‐inflammation that damages oligodendrocytes and myelin sheaths of the axons,^2^ differences in neuroinflammation extent between MPS IH and MPS IA have not been reported in vivo.

While chemotherapy (CHT) administered before HSCT represents an additional source of WM alteration in the early stage of brain development in MPS IH patients, those who underwent early HSCT exhibited lower FWF in frontal WM and higher (i.e., “better”) FA^40^ in CC and parietal WM. CHT treatment has been associated with structural and functional brain disturbances in various patient populations.^41^, ^42^, ^43^ Animal models showed that myelin and cognitive alterations seen after CHT are likely the result of persistent neuroinflammation.^44^ The temporal evolution of myelination progresses from posterior to anterior and inferior to superior brain parts, leading to the relative vulnerability of anterior WM areas to pathological influences in leukemia survivors.^41^ While adverse effects of CHT on the WM are congruently shown among various patient populations^41^, ^43^ and may partially affect WM in MPS IH, relationships between FA and FWF showed positive effects of early HSCT on the CC, frontal, and parietal WM, likely due to later myelination in these topographically specific brain areas. Thus, our results align with previous studies in MPS IH that demonstrated the maximal benefits of HSCT when performed early in life.^45^ Nevertheless, cross‐sectional analysis cannot sufficiently evaluate various impacts of CHT and MPS disease, and longitudinal studies in young MPS patients are warranted.

Similar to post‐CHT outcomes,^41^, ^42^ cognitive and attention deficits were altered in MPS IH. While MPS IH patients showed lower scores in all neuropsychological measures, they significantly underperformed in responding speed consistency and inattention,^31^ thus demonstrating a more profound alteration of neuropsychological measurements in MPS IH than in MPS IA.

The analyses between MPS IA and HC showed distinctions in the spatial distribution of WM alteration compared to MPS IH deficits. In general, the MPS IA subtype is characterized by later onset, slower progression of brain alteration, and distinctive severity of cognitive and attention deficits.^15^ The variance in cognitive performance is partially caused by ERT, which is used as a preferential treatment strategy in MPS IA. Although ERT sufficiently deals with somatic disease, the blood–brain barrier limits the penetration of ERT to the brain with no or minimal treatment efficacy.^46^, ^47^ MPS IA showed significantly higher MD underpinned by higher RD in cingulate WM and higher FWF in cingulate and temporal WM in MPS IA relative to HC. Temporal and cingulate results align with a recent DTI canine study that demonstrated higher FA in several WM regions of one MPS dog.^6^

Exploratory regression analyses revealed that attention performance measured as TOVA subscales correlated with diffusion measures in frontal and parietal WM in MPS IH, while in MPS IA, attention was related to temporal and central WM. In MPS, quantitative brain MRI studies previously investigated the links between brain volumes and TOVA‐derived attention measures.^48^, ^49^ Cerebral WM and CC volumes correlated with attention in mucopolysaccharidosis type II (Hunter syndrome) patients with an attenuated phenotype.^48^ Similarly, the lower attention outcomes were associated with lower CC volume in MPS IH and MPS IA patients.^49^ The findings further confirm the association between WM alteration, even quantified at the macrostructural level, and impaired attention.

Parietal and frontal WM have been known to be involved in attention,^50^ with the temporal region also suggested as a critical hub for attention.^51^ The temporal and cingulate areas, interconnected through the cingulum bundle, were indeed confirmed critical attention hubs in schizophrenia,^52^ healthy older individuals,^53^ or bipolar disorder.^54^, ^55^ Thus, our findings imply spatial distinctions in attention processing between MPS subtypes. A previous study found a relationship between CC volumes, FA, and attention,^12^ but we did not detect any relationship between attention and CC. It is potentially due to the methodological approach since we accounted for the atrophy level and the number of PVS spaces in our DTI metrics calculations. Indeed, a previous study reported an association between PVS and mental deficits in MPS.^20^ The involvement of distinct brain regions in attention in MPS IH and IA is likely caused by phenotypical and treatment‐strategy differences.

An analysis of morphologically abnormal brains required ROI analysis, which allowed to remain in a subject's space without the need to normalize images to a standard brain template/space, as required by voxel‐based morphometry or tract‐based spatial statistics analysis methods.^56^ A normalization procedure may shift the anatomical position due to misregistration, especially in MPS I patients presenting with atrophy and ventricular size/position differences caused by the macrostructural brain changes.^56^

The spatial distinctions of FWF between MPS IH and MPS IA confirmed the importance of FWC in DTI analysis in MPS patients. Recently, FWC helped to delineate subtle disease‐related changes in stress‐related depression,^57^ schizophrenia,^4^ and Parkinson's disease^58^, ^59^ or treatment‐related side effects.^60^ The existence of the enlarged PVS further potentiates the need for an FWC approach in MPS. FWC helps to depict tissue changes, likely caused by demyelination, as a key mechanism of WM alteration in MPS IH and implies region‐specific involvement of distinct microstructural mechanisms in WM damage in MPS IH and MPS IA. Overall, the significant increase of FWF in MPS IH, with limited regional FWF changes in MPS IA, suggests a more profound level of edema or inflammation in WM alteration of MPS IH patients. FWC revealed underlying WM microstructural changes in MPS subtypes that would be otherwise masked by pathologically increased extracellular water content. Thus, FWF is a unique measure for longitudinal MPS studies to monitor disease progression and treatment effects.

### 4.1 Limitations

The between‐group age imbalance required a sample size reduction applied in post hoc cross‐sectional analyses. The diffusion acquisition only included a single shell of diffusion‐weighted images to limit the MRI acquisition time in this young and unique population. With newer multi‐slice technology, additional diffusion shells can be collected at a similar acquisition time to provide a more robust fit of the free‐water model.^61^ One of the challenges of incorporating DTI into routine clinical practice is the time required for image acquisition, as standard DTI scans might take 5–20 min depending on the resolution and the number of diffusion directions being acquired.^40^ Also, standardized protocols and normative data, together with efficient integration of DTI into the clinical workflow, are critical to ensure consistency and reliability of DTI metrics across different MRI institutions.^40^ Advances in diffusion imaging, such as multi‐band imaging and simultaneous multi‐slice acquisition, reduce scan times,^62^ making DTI more feasible for clinical use. Scanning time is critical in patients unable to stay still within the scan acquisition (e.g., due to very young age or intellectual disability) and, thus, requiring sedation or general anesthesia.^63^ In MPS, airway management before general anesthesia poses a high risk of complicated or failed tracheal intubation in more than 40% and 12% of cases, respectively.^63^, ^64^ Moreover, 20% of deaths associated with surgery in MPS I were directly related to airway obstruction or difficult intubations.^65^ Future MRI protocols for MPS assessing WM integrity should rely on advanced diffusion imaging techniques to mitigate the health risks of the MRI procedure.

## 5 CONCLUSION

The FWC analysis effectively delineates MPS‐related WM deficits without unwanted extracellular effects such as atrophy, PVS, and potential edema or neuro‐inflammation. Our results revealed that differences in the severity and spatial distribution of WM alteration between the MPS IH and MPS IA subtypes are best reflected by the FWF and RD measures, likely reflecting the severity of extracellular and cellular WM changes, respectively. WM parameters were related to the age at HSCT in MPS IH patients and attention performance in both MPS subtypes, with distinctions found in the spatial organizations of the WM regions preferentially associated with attention performance. Hence, our study established the importance of the FWC DTI analysis approach to address MPS‐related morphological brain abnormalities, making it a promising approach for future longitudinal trials.

## AUTHOR CONTRIBUTIONS

Alena Svatkova designed the MRI data analysis, analyzed and interpreted the brain MRI data, designed tables and figures, drafted the first manuscript, revised the manuscript for important intellectual content, and approved the final version for publication. Ofer Pasternak developed the free‐water correction of MRI data, revised the manuscript for important intellectual content, and approved the final version for publication. Kyle D. Rudser provided guidance on the statistical analysis, revised the manuscript for important intellectual content, and approved the final version for publication. Petr Bednarik interpreted the MRI data, revised the manuscript for important intellectual content, and approved the final version for publication. Bryon A. Mueller designed the MRI protocol, provided guidance on the MRI analysis, revised the manuscript for important intellectual content, and approved the final version for publication. Julie B. Eisengart interpreted the neuropsychological data, revised the manuscript for important intellectual content, and approved the final version for publication. Kathleen A. Delaney acquired and analyzed the neuropsychological data, revised the manuscript for important intellectual content, and approved the final version for publication. Elsa G. Shapiro conceptualized and designed the overall study, interpreted the neuropsychological data and the relationship between neuropsychological and MRI data, revised the manuscript for important intellectual content, and approved the final version for publication. Chester B. Whitley conceptualized the study, revised the manuscript for important intellectual content, and approved the final version for publication. Igor Nestrasil conceptualized and designed the study, analyzed and interpreted the data, revised the manuscript for important intellectual content, and approved the final version for publication.

## FUNDING INFORMATION

The study was funded by the Sanofi Genzyme (GZ‐2014‐11 270), the National MPS Society, Million Dollar Bike Ride from the University of Pennsylvania (MDBR‐16‐125‐MPS, MDBR‐15‐214‐MPS, 303052MPSI‐16‐003‐02), the Ryan Foundation, the Rare Diseases Clinical Research Network, Lysosomal Disease Network, NIH U54NS065768, and the resources of the CMRR (supported by NIBIB P41 EB027061, P30 NS076408, and 1S10OD017974–01). The HC were recruited in the study funded by Shire/Takeda (4–7 control study). Alena Svatkova has received funding from the European Union's Horizon 2020 research and innovation programme under the Marie Skłodowska‐Curie grant (Agreement No. 794986).

## CONFLICT OF INTEREST STATEMENT

Alena Svatkova reports no disclosures. Ofer Pasternak reports no disclosures. Kyle D. Rudser reports no disclosures. Petr Bednarik reports no disclosures. Bryon A. Mueller reports no disclosures. Julie B. Eisengart, Research support from Lysogene, Sangamo, Shire/Takeda and Sobi; consulting fees from ArmaGen, Denali Therapeutics, JCR Pharmaceutical, Orchard Therapeutics, ReGenXBio and Shire/Takeda; and advisory boards for Amicus Therapeutics, bluebird bio, Denali, Orchard Therapeutics, ReGenXBio, Sanofi Genzyme and Shire/Takeda. Kathleen A. Delaney is an employee of BioMarin Pharmaceutical Inc. Elsa G. Shapiro is a consultant for Shapiro Neuropsychology Consulting LLC. Chester B. Whitley received Research support from the National Institutes of Health, including Lysosomal Disease Network (RDCRN) NIH U54NS065768. Igor Nestrasil, Research support from the Million Dollar Bike Ride—University of Pennsylvania, National MPS Society, BioMarin, Sanofi Genzyme, and Shire/Takeda. Consultant for ICON, Bioclinica/Clario, and Quantims.

## ETHICS STATEMENT

Study participants were recruited in the University of Minnesota IRB‐approved studies (IRB protocol ID 0905M65804—PI Shapiro, STUDY00014153—PI Whitley, 1202M10383—PI Nestrasil).

## PATIENT CONSENT STATEMENT

All participants, parents, and/or legal guardians gave signed informed consent; assent was obtained from children and those over 18 years with a legal guardian, which included permission to share de‐identified data with the Rare Disease Clinical Research Network (RDCRN) Data Monitoring and Coordination Center for patients.

## Supporting information


**Supplementary Table S1.** Summarizes outcomes of the linear regression analysis in MPS IH subjects that underwent hematopoietic stem cell transplant (HSCT). The models use FWC‐corrected diffusion and FWF values for all analyzed regions as the dependent variables, with age at HSCT and actual age (i.e., age at the time of the scan) as independent variables. The standardized coefficients (Beta) indicate the strength and direction of the relationship between independent and dependent variables, along with p‐values (p), where statistical significance is considered at *p* < 0.01. The table also provides the unstandardized coefficient (Slope) showing how much the dependent variable changes for each unit increase in the independent variable, with the lower and upper bounds of the 95% confidence intervals (CI). Additionally, the table presents the correlation coefficient, the proportion of variance (*R* ^2^) explained by the independent variables, and the p‐values for the overall model.

## Data Availability

De‐identified individual participant data that underlie the results reported in this article can be shared upon reasonable request from researchers who provide a methodologically sound proposal to the corresponding author. The data can be shared only for study participants who agreed with and consented to the sharing of de‐identified data in future studies.
