# Supplementary material for: Diffusion tensor imaging with free‐water correction reveals distinctions between severe and attenuated subtypes in Mucopolysaccharidosis type I
Source: J Inherit Metab Dis. 2025 Jan 6;48(1):e12830. doi: 10.1002/jimd.12830 (PMC11703597; doi:10.1002/jimd.12830)
Supplement: Supplementary file 1 — Supplementary Table S1. Summarizes outcomes of the linear regression analysis in MPS IH subjects that underwent hematopoietic stem cell transplant (HSCT). The models use FWC‐corrected diffusion and FWF values for all analyzed regions as the dependent variables, with age at HSCT and actual age (i.e., age at the time of the scan) as independent variables. The standardized coefficients (Beta) indicate the strength and direction of the relationship between independent and dependent variables, along with p‐values (p), where statistical significance is considered at p < 0.01. The table also provides the unstandardized coefficient (Slope) showing how much the dependent variable changes for each unit increase in the independent variable, with the lower and upper bounds of the 95% confidence intervals (CI). Additionally, the table presents the correlation coefficient, the proportion of variance (R 2) explained by the independent variables, and the p‐values for the overall model. [file JIMD-48-0-s001.docx]

|  |  | **Age at HSCT** | | | | | **Actual age** | | | | | **Model** | | |
| --- | --- | --- | --- | --- | --- | --- | --- | --- | --- | --- | --- | --- | --- | --- |
|  | **Region** | **Beta** | **p** | **Slope** | **95%CI** | | **Beta** | **p** | **Slope** | **95%CI** | | **R** | **R²** | **p** |
|  |  |  |  |  | **lower** | **upper** |  |  |  | **lower** | **upper** |  |  |  |
| **AD** | **CC** | -0.37 | 0.10 | -60.2 | -134.3 | 13.8 | 0.16 | 0.47 | 3.8 | -7.1 | 14.7 | 0.42 | 0.18 | 0.17 |
|  | **Central** | -0.04 | 0.88 | -4.1 | -59.4 | 51.2 | 0.22 | 0.36 | 3.6 | -4.5 | 11.8 | 0.23 | 0.05 | 0.63 |
|  | **Cingulate** | -0.08 | 0.75 | -8.3 | -63.0 | 46.1 | 0.00 | 0.99 | -0.03 | -8.0 | 8.0 | 0.08 | 0.01 | 0.95 |
|  | **Frontal** | -0.17 | 0.47 | -11.7 | -45.0 | 21.6 | -0.18 | 0.45 | -1.8 | -6.7 | 3.1 | 0.23 | 0.05 | 0.62 |
|  | **Parietal** | -0.49 | 0.02 | -39.5 | -73.3 | -5.7 | 0.19 | 0.34 | 2.3 | -2.7 | 7.3 | 0.55 | 0.31 | 0.04 |
|  | **Temporal** | -0.25 | 0.30 | -19.7 | -58.5 | 19.2 | 0.03 | 0.89 | 0.4 | -5.4 | 6.1 | 0.25 | 0.06 | 0.55 |
| **FA** | **CC** | -0.56 | **0.007*** | -50.6 | -85.7 | -15.5 | -0.39 | 0.05 | -5.2 | -10.3 | 0.0 | 0.63 | 0.40 | 0.01 |
|  | **Central** | -0.19 | 0.37 | -12.0 | -39.7 | 15.7 | 0.45 | 0.04 | 4.3 | 0.2 | 8.4 | 0.52 | 0.27 | 0.06 |
|  | **Cingulate** | -0.49 | 0.03 | -28.4 | -53.5 | -3.2 | -0.16 | 0.46 | -1.3 | -5.1 | 2.4 | 0.49 | 0.24 | 0.08 |
|  | **Frontal** | -0.46 | 0.03 | -17.9 | -33.4 | -2.3 | 0.30 | 0.14 | 1.7 | -0.6 | 4.0 | 0.59 | 0.35 | 0.02 |
|  | **Parietal** | -0.62 | **0.002*** | -27.5 | -43.3 | -11.7 | 0.25 | 0.16 | 1.6 | -0.7 | 4.0 | 0.70 | 0.49 | **0.002*** |
|  | **Temporal** | -0.48 | 0.02 | -18.9 | -35.0 | -2.8 | 0.24 | 0.23 | 1.4 | -1.0 | 3.8 | 0.57 | 0.33 | 0.03 |
| **FWF** | **CC** | 0.22 | 0.28 | 13.7 | -11.8 | 39.2 | 0.55 | 0.01 | 5.0 | 1.2 | 8.8 | 0.56 | 0.32 | 0.03 |
|  | **Central** | 0.22 | 0.32 | 7.5 | -7.8 | 22.8 | -0.33 | 0.14 | -1.6 | -3.9 | 0.6 | 0.42 | 0.18 | 0.17 |
|  | **Cingulate** | 0.49 | 0.03 | 19.2 | 2.1 | 36.3 | 0.12 | 0.58 | 0.7 | -1.9 | 3.2 | 0.49 | 0.24 | 0.09 |
|  | **Frontal** | 0.38 | 0.048 | 12.1 | 0.1 | 24.2 | -0.50 | 0.01 | -2.4 | -4.1 | -0.6 | 0.67 | 0.45 | **0.005*** |
|  | **Parietal** | 0.33 | 0.16 | 7.9 | -3.4 | 19.1 | -0.12 | 0.61 | -0.4 | -2.1 | 1.2 | 0.36 | 0.13 | 0.28 |
|  | **Temporal** | 0.42 | 0.04 | 9.7 | 0.7 | 18.7 | -0.40 | 0.04 | -1.4 | -2.7 | -0.03 | 0.63 | 0.39 | 0.01 |
| **MD** | **CC** | 0.08 | 0.72 | 8.3 | -39.2 | 55.8 | 0.45 | 0.05 | 7.1 | 0.1 | 14.1 | 0.45 | 0.20 | 0.14 |
|  | **Central** | 0.02 | 0.93 | 1.1 | -25.8 | 28.1 | -0.02 | 0.92 | -0.2 | -4.2 | 3.8 | 0.03 | 0.001 | 0.99 |
|  | **Cingulate** | 0.28 | 0.23 | 20.3 | -14.0 | 54.3 | 0.17 | 0.47 | 1.8 | -3.2 | 6.8 | 0.31 | 0.10 | 0.41 |
|  | **Frontal** | -0.03 | 0.90 | -1.5 | -25.2 | 22.2 | -0.27 | 0.25 | -2.0 | -5.5 | 1.5 | 0.27 | 0.07 | 0.51 |
|  | **Parietal** | -0.35 | 0.12 | -19.0 | -43.4 | 5.3 | 0.18 | 0.42 | 1.4 | -2.2 | 5.0 | 0.42 | 0.18 | 0.17 |
|  | **Temporal** | -0.18 | 0.46 | -9.7 | -36.9 | 17.5 | -0.06 | 0.80 | -0.5 | -4.5 | 3.5 | 0.18 | 0.03 | 0.75 |
| **RD** | **CC** | 0.36 | 0.08 | 42.6 | -5.3 | 90.6 | 0.50 | 0.02 | 8.7 | 1.6 | 15.8 | 0.58 | 0.33 | 0.03 |
|  | **Central** | 0.09 | 0.68 | 3.8 | -15.1 | 22.6 | -0.35 | 0.14 | -2.1 | -4.8 | 0.7 | 0.37 | 0.14 | 0.26 |
|  | **Cingulate** | 0.47 | 0.03 | 34.6 | 3.0 | 66.2 | 0.25 | 0.24 | 2.7 | -1.9 | 7.4 | 0.50 | 0.25 | 0.07 |
|  | **Frontal** | 0.08 | 0.71 | 3.5 | -16.1 | 23.1 | -0.34 | 0.15 | -2.1 | -5.0 | 0.8 | 0.36 | 0.13 | 0.29 |
|  | **Parietal** | -0.20 | 0.39 | -8.6 | -29.4 | 12.1 | 0.15 | 0.53 | 0.9 | -2.1 | 4.0 | 0.27 | 0.07 | 0.51 |
|  | **Temporal** | -0.11 | 0.66 | -4.7 | -26.6 | 17.2 | -0.14 | 0.56 | -0.9 | -4.1 | 2.3 | 0.16 | 0.03 | 0.79 |

**Supplementary Table 1**. Summarizes outcomes of the linear regression analysis in MPS IH subjects that underwent hematopoietic stem cell transplant (HSCT). The models use FWC-corrected diffusion and FWF values for all analyzed regions as the dependent variables, with age at HSCT and actual age (i.e., age at the time of the scan) as independent variables. The standardized coefficients (Beta) indicate the strength and direction of the relationship between independent and dependent variables, along with p-values (p), where statistical significance is considered at p < 0.01. The table also provides the unstandardized coefficient (Slope) showing how much the dependent variable changes for each unit increase in the independent variable, with the lower and upper bounds of the 95% confidence intervals (CI). Additionally, the table presents the correlation coefficient, the proportion of variance (R²) explained by the independent variables, and the p-values for the overall model.
